# Integrative analysis reveals novel associations between DNA methylation and the serum metabolome of adolescents with type 2 diabetes: A cross-sectional study

**DOI:** 10.3389/fendo.2022.934706

**Published:** 2022-10-10

**Authors:** Prasoon Agarwal, Brandy A. Wicklow, Allison B. Dart, Nikho A. Hizon, Elizabeth A.C. Sellers, Jonathan M. McGavock, Charlotte P. J. Talbot, Mario A. Fonseca, Wayne Xu, James R. Davie, Meaghan J. Jones, Animesh Acharjee, Vernon W. Dolinsky

**Affiliations:** ^1^ Diabetes Research Envisioned and Accomplished in Manitoba (DREAM), Research Theme of the Children’s Hospital Research Institute of Manitoba, University of Manitoba, Winnipeg, MB, Canada; ^2^ Department of Pharmacology and Therapeutics, University of Manitoba, Winnipeg, MB, Canada; ^3^ Department of Pediatrics and Child Health, University of Manitoba, Winnipeg, MB, Canada; ^4^ Department of Biochemistry and Medical Genetics, University of Manitoba, Winnipeg, MB, Canada; ^5^ Research Institute in Oncology and Hematology, University of Manitoba, Winnipeg, MB, Canada; ^6^ Institute of Cancer and Genomic Sciences, University of Birmingham, Winnipeg, MB, Canada; ^7^ Institute of Translational Medicine, University Hospitals Birmingham National Health Service (NHS) Foundation Trust, Birmingham, United Kingdom; ^8^ National Institute for Health and Care Research (NIHR) Surgical Reconstruction and Microbiology Research Centre, Birmingham, United Kingdom

**Keywords:** type 2 diabetes mellitus, metabolomics, DNA methylation, integration of data, pediatrics

## Abstract

**Objective:**

Rates of type 2 diabetes (T2D) among adolescents are on the rise. Epigenetic changes could be associated with the metabolic alterations in adolescents with T2D.

**Methods:**

We performed a cross sectional integrated analysis of DNA methylation data from peripheral blood mononuclear cells with serum metabolomic data from First Nation adolescents with T2D and controls participating in the Improving Renal Complications in Adolescents with type 2 diabetes through Research (iCARE) cohort study, to explore the molecular changes in adolescents with T2D.

**Results:**

Our analysis showed that 43 serum metabolites and 36 differentially methylated regions (DMR) were associated with T2D. Several DMRs were located near the transcriptional start site of genes with established roles in metabolic disease and associated with altered serum metabolites (e.g. glucose, leucine, and gamma-glutamylisoleucine). These included the free fatty acid receptor-1 *(FFAR1*), upstream transcription factor-2 (*USF2*), and tumor necrosis factor-related protein-9 (*C1QTNF9*), among others.

**Conclusions:**

We identified DMRs and metabolites that merit further investigation to determine their significance in controlling gene expression and metabolism which could define T2D risk in adolescents.

## Introduction

Type 2 diabetes (T2D) is a global epidemic, but a major concern is the rising incidence among youth (1). In 1990 T2D accounted for only 3% of new diagnoses of diabetes among U.S. children, but by 2010 that number rose to 45% in some populations (2). In the Canadian province of Manitoba, the annual incidence of T2D in children increased from 22.8 to 35.7 cases per 100,000 children between 2007 and 2017, affecting a disproportionate number of First Nations youth (3). An understanding of the underlying pathology of T2D is paramount to improving clinical outcomes.

Adult-onset T2D progresses gradually from impaired glucose tolerance to β-cell failure, but in youth, the loss of β-cell function is accelerated (4). Single gene polymorphisms alone do not explain the rapid rise of T2D in youth observed over a single generation, suggesting the pathophysiology of T2D in youth could involve additional gene and environment interactions (5). As our study population is First Nations youth in Canada, we remain cognizant of the environmental, social and political impact of colonization which has had a powerful detrimental effect on Indigenous populations. Colonization disrupted food sovereignty and connection to the land that blocked access to traditional farming, food-gathering, hunting and fishing practices that are fundamental to the maintenance of health within First Nations populations and an associated dependence on non-traditional foods with inferior nutrient qualities. Environmental alterations such as these could have a major influence on the epigenome (changes occurring on the DNA where the DNA sequence itself is not changed), which then may affect T2D risk (6, 7).

Metabolites play a key role as both biomarkers and mediators of T2D development, and metabolic perturbations could explain the aggressive course of T2D in youth (8, 9). Though studies have characterized the circulating metabolomic profile of adults with insulin resistance and T2D, data from pediatric populations are limited. Some studies reported that aromatic and branched chain amino acids (BCAAs) were associated with insulin resistance and T2D in youth (8, 10–13). Through a combination of indirect calorimetry and mass spectrometry researchers showed that, unlike adults with T2D, changes in acylcarnitine’s and fatty acid oxidation were not observed in youth with T2D (8). Collectively these findings suggest that the metabolic perturbations of T2D are different in adolescents compared to adults. Growing evidence suggests that epigenetic modifications including DNA methylation can be affected by the nutritional and metabolic state (14). Persistent changes in the methylome could also be associated with pathogenic metabolic profiles. For example, alterations in DNA methylation have been reported in peripheral blood mononuclear cells (PBMCs) and human islets from T2D adults (7). To date, no studies have linked DNA methylation and serum metabolomic profiling of adolescents diagnosed with T2D.

Since the pathogenesis of pediatric T2D is different from T2D in adults, the objective of this study was to explore changes in the epigenetic landscape of peripheral blood mononuclear cells that are associated with an altered serum metabolome. In this study, we linked differentially methylated regions (DMRs) to five biologically important metabolites that were significantly altered in adolescents with T2D. The metabolites correlated with several DMRs in adolescents with T2D that were located near the transcriptional start sites (TSS) of several biologically relevant genes, including the free fatty acid receptor-1 *(FFAR1*), upstream transcription factor-2 (*USF2*), and the novel cytokine, tumor necrosis factor-related protein-9 (*C1QTNF9*). These data will help us generate new hypotheses to investigate the mechanisms that influence the metabolomic profile of adolescents diagnosed with T2D.

## Material and methods

### iCARE cohort

The iCARE cohort study (15) received informed consent from study participants and approval from the University of Manitoba/Health Sciences Centre Research Ethics Board (HS13255), First Nations patient and parent advisory committee and the First Nation Health and Social Secretariat of Manitoba and the iCARE participant and parent advisory committees. In this study, we performed a subgroup analysis of the iCARE cohort that consisted of 12- to 24-year-old First Nations adolescents diagnosed with T2D prior to 18 years of age (mean age of 15). The controls are normoglycemic overweight or obese adolescents at risk of developing T2D (mean age of 16). All of the patients were fasting a minimum of 8h prior to sample collection. The diagnosis of T2D was based on biochemical and clinical criteria and the absence of insulin and glutamic acid decarboxylase antibodies. All the clinical parameters are shown in **Table 1** (for the whole cohort) and **Supplementary Table 3** (samples selected from the cohort for SOLiD sequencing). Non-adjusted p-values were calculated using Student’s t- test and Chi-Square test.

**Table 1:** Anthropometric characteristics of the primary cohort used in the study.

Anthropometric characteristics	Control adolescents (n=42)	Adolescents with T2D (n=113)	P-value	Statistical test
Age (years)	16.08 (3.11)	15.24 (2.58)	0.09	t-test
Gender (Male/Female)	(14/28)	(35/78)	0.78	Chi-Square
Weight (kg)	90.33 (23.56)	86.46 (22.73)	0.35	t-test
Height (cm)	166.00 (8.53)	165.28 (9.64)	0.67	t-test
Waist (cm)	104.96 (19.72)	105.03 (18.28)	0.98	t-test
BMI (kg/m²)	32.43 (6.79)	31.37 (6.49)	0.37	t-test
BMI Z-score	1.66 (0.83)	1.79 (0.72)	0.31	t-test
Duration of diabetes (years)	/	2.12 [3.12]	/	/
Albuminuria (%)	9.5	39.6	<0.001	Chi-Square
Ambulatory Hypertension (%)	22.5	22.8	0.92	Chi-Square
Nocturnal Hypertension (%)	22.5	32.7	0.23	Chi-Square
Plasma glucose (mmol/L)	3.44 (1.59)	11.38 (5.95)	<0.001	t-test
ALT (units/L)	25.64 (23.05)	29.28 (22.28)	0.32	t-test
AST (units/L)	22.61 (10.35)	22.45 (14.91)	0.95	t-test
HbA1c (mmol/mol)	5.65 (0.24)	9.38 (2.73)	<0.001	t-test
Total Cholesterol (mmol/L)	3.91 (0.67)	4.45 (0.98)	<0.01	t-test
Triglycerides (mmol/L)	1.28 (0.64)	2.19 (2.13)	<0.01	t-test
HDL (mmol/L)	1.22 (0.31)	1.12 (0.28)	0.06	t-test
LDL (mmol/L)	2.10 (0.51)	2.37 (0.68)	<0.05	t-test
Total Cholesterol/HDL Ratio	3.37 (0.93)	3.95 (1.19)	<0.05	t-test
LDL/HDL Ratio	1.83 (0.64)	2.17 (0.79)	<0.05	t-test

Values are means (SD). For the variable “duration of diabetes”, values are presented as median [interquartile range]. P values ≦ 0.05 were considered significant. P-values were calculated using Student’s t-test and Chi-Square test.

T2D, type 2 diabetes mellitus; BMI, body mass index; ALT, alanine aminotransferase; AST, aspartate aminotransferase; HbA1c, glycated hemoglobin. There are 42 controls and 113 patients with T2D included in this study.

### Metabolomics analysis of T2D adolescent serum

Samples were prepared using the automated MicroLab STAR^®^ system (Hamilton Company, Boston, U.S.A). Metabolomic methods utilized a Waters ACQUITY ultra-performance liquid chromatography (UPLC) and a Thermo Scientific Q-Exactive high resolution/accurate mass spectrometer interfaced with a heated electrospray ionization (HESI-II) source and Orbitrap mass analyzer operated at 35,000 mass resolution. [See **Supplementary Methods (Supplement S1)**]. Raw data was extracted, peak-identified and QC processed using Metabolon’s hardware and software. Compounds were identified by comparison to library entries of purified standards or recurrent unknown entities. Entities with more than 20% missing values were removed, MetaboAnalyst 4.0 was used to impute values (replace the value by a small value which is half of the minimum positive value in the original data), followed by data filtering and auto scaling. Details of metabolite quantification and data scaling are found in the **Supplementary Methods (Supplement S1)**.

### SOLiD library preparation and bioinformatics analysis

Genomic DNA was extracted from the peripheral blood mononuclear cells (PBMC) from a subset of the cohort that were included in the serum metabolomics dataset and was comprised of 21 adolescents with T2D and 10 control participants. Libraries were prepared according to the MethylMiner™ manufacturer’s protocol (ThermoFisher scientific Catalog number ME10025). MethylMiner enriches double-stranded methylated DNA based on CpG methylation density, with increased sensitivity over antibody-based methods. The methylated DNA obtained was subjected to SOLiD sequencing where 50-bp single end sequence reads were ensured by quality check (noise to signal ratio). The sequence reads were mapped to the human reference genome (hg19) using the MethylMiner™ Mapping Analysis module of the LifeScope v2.5.1 software package (Life Technologies).

### Data preprocessing and normalization

Regions enriched with DNA methylation across the genome were identified using the “callpeak” function from Model-based Analysis of ChIP-seq (MACS2) (16), with model fold = [5, 30] and FDR < 0.05 on the aligned reads generating 31 peaksets. The below peak filtering was done using the *DiffBind* package in R (17). As we included males and females in the cohort, we removed sex chromosomes using ENCODE blacklist regions (18) and those present in at most 2 samples were excluded from downstream analyses, leaving a total of 732 984 peaks. The *edgeR* package (19) was used to normalize for sequencing depth and effective library size by transforming data into counts per million and performing the trimmed mean of M-value (TMM) normalization.

### Linear modelling

Cell-type proportion effects were corrected for using the *sva* package (20) reference-free cell-type correction method. The top 2 surrogate variables were included in the regression model. A generalized linear model in *edger* was used to identify differentially methylated regions (DMRs) between diabetes cases and controls using the following formula: Reads ~ Diabetes status + Age + Sex + BMI + SVs. Multiple testing was corrected for using the Benjamini-Hochberg method. Statistical significance was set at FDR < 0.05.

### Multivariate statistical analysis

The multiomic data sets (metabolomic and DNA methylation data) were analyzed using the multivariate statistical analysis tools found in SIMCA (version 13; Umetrics AB, Umeå, Sweden) and Metaboanalyst 5.0. Unsupervised hierarchical clustering was performed for the 43 statistically significant metabolites to identify different metabolite clusters. Based on the relevance of metabolites to the profile of T2D patients from other studies, we selected five metabolites from each of the separate clusters for data fusion (gamma-glutamylisoleucine, glucose, leucine, palmitoylcholine and sphingomyelin). We used a supervised classification method called orthogonal partial least-squares discriminant analysis (OPLS-DA) to identify the metabolites and DMRs that are most interesting for this analysis. We quantified model statistics based on the fraction of the sum of squares for the selected component (*R* ^2^), which equates to the percentage of the model variance explained, and the predictive ability (*Q* ^2^). Cross-validation was performed to predict and estimate the model performance (whether models were over fitted). For OPLS-DA models, random permutation was used whereby the class membership of individual samples are permuted randomly. In addition, ANOVA of the cross-validated residuals (CV-ANOVA) test was performed within Simca to further validate the models validated by selecting two thirds of the samples randomly and then predicting the class membership of the rest of the one third. We used variable importance in the projection scores (VIP) to prioritize the metabolites. A VIP score cutoff of >1.5 was considered in the model (21).

### Peak annotation and omics data fusion and visualization

The peaks were annotated to -5000 bp to 5000 bp of the (TSS of the nearest gene) the nearest gene using the software Genomic region enrichment of Annotation s tool (GREAT v 4.04) where human genome assembly hg19 was used for annotation and for the background the whole genome was used. Omics data fusion was performed on selected metabolites and the peaks that were annotated to the closest genes. The selected metabolites and DMRs were fused based on the Pearson correlation values and visualized *via* R statistical software (https://www.r-project.org/) package called qgraph (22). IGV v.2.13.2 was used to make the genomic track where the BAM files for all the controls and T2D samples were used. All of the tracks were auto scaled.

## Results

### Patient characteristics

We used a cross sectional design to compare the serum metabolomic profiles of 113 First Nations adolescents (age range 10 - 24 years and BMI range of 19 – 48 kg/m^2^) with T2D to 42 normoglycemic First Nations controls. Higher levels of fasting blood glucose and HbA1c were observed in the adolescents with T2D compared to controls (**Table 1**; p-value <0.001 using t-test). Among adolescents with T2D, the average time from diagnosis of T2D was 2.12 years. HbA1c was associated with weight (r=-0.20), waist circumference (r=-0.24) and BMI z-score (r=-0.31); however, in controls HbA1c was associated with ALT (r=0.32) (**Supplementary Figure 2**).

### Metabolites associated with T2D in adolescents

**Figure 1:**
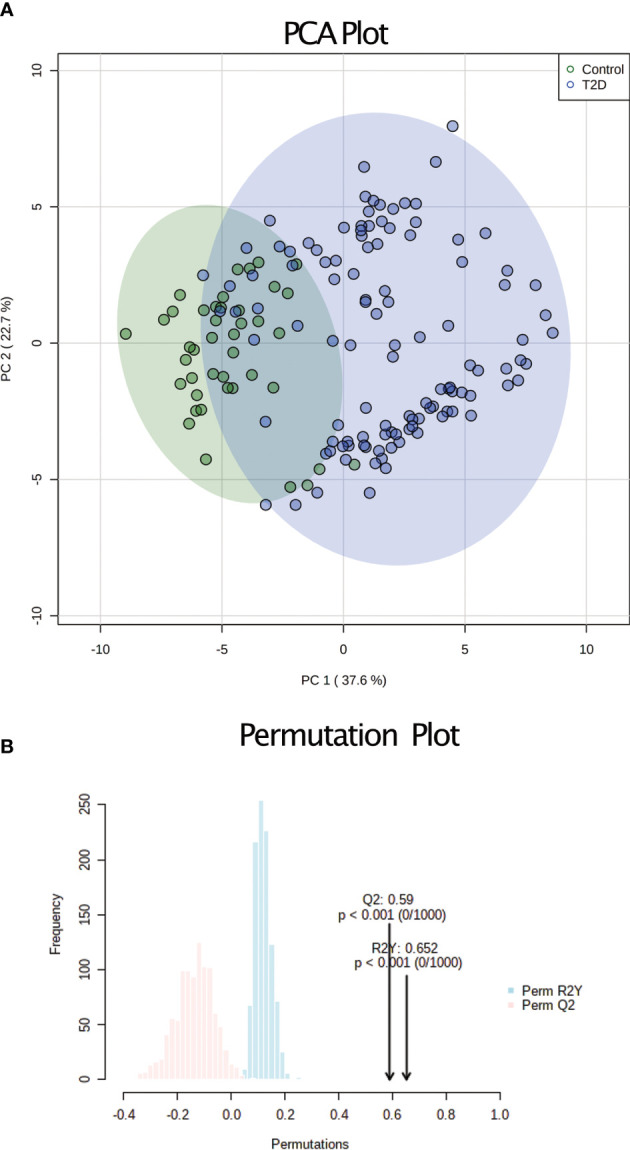
Unsupervised analysis of serum metabolomics. **(A)** PCA analysis based on the 43 metabolites and all 155 samples. The controls are shown in green and the T2D samples in blue **(B)** Permutation conducted to validate the variation obtained during the PCA. The R2 (shown in blue) and Q2 (shown in red) values indicate the robustness of the PCA model.

**Figure 2:**
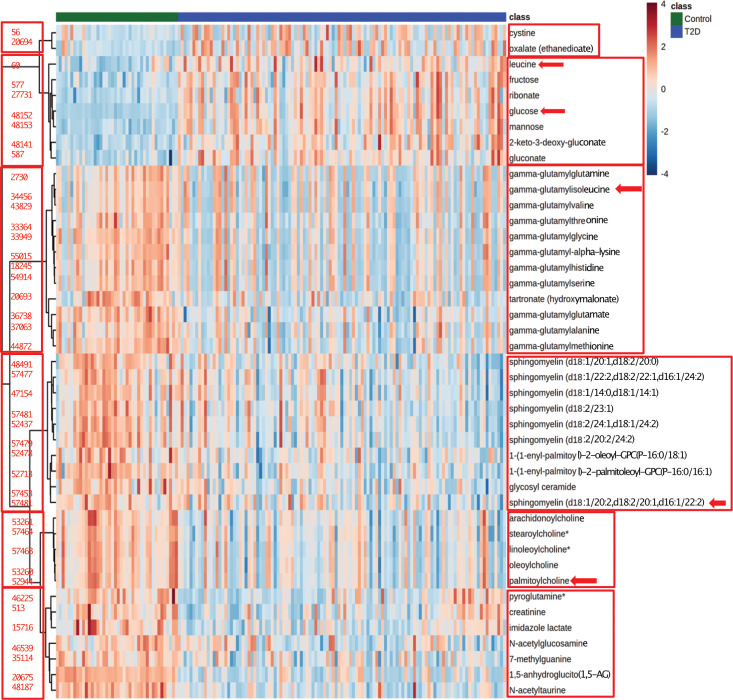
Heatmap of differential levels of 43 serum metabolites. 43 metabolites that were significantly different between T2D adolescents (blue color class) and control adolescents (green color class). Red color indicates the increased and the blue indicates reduced levels. The red boxes show the six major clusters formed. On the left of cluster is shown the compound identification for the respective metabolite. The red arrows show the five metabolites that were used for data integration.

**Figure 3:**
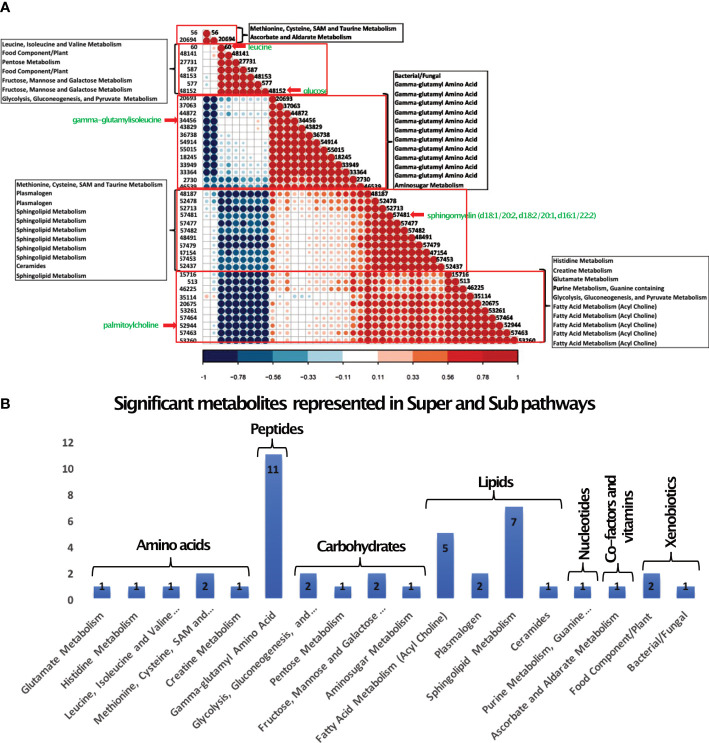
Pathway analysis of Serum Metabolites. **(A)** Pearson correlation-based clustering of the significant metabolites. Five major clusters obtained are shown in red boxes. Each metabolite is represented by their compound identification (**Table 2** shows the respective metabolites). The sub pathways of the metabolites are represented in the boxes. The five metabolites chosen for data integration are indicated by a red arrow. **(B)** 43 metabolites were categorized into seven super pathways, including lipids (35%), peptides (26% gamma-glutamyl amino acids), amino acids (10%), carbohydrates (17%), nucleotide (2% purine metabolism), cofactors and vitamins (2% ascorbate and aldarate metabolism), and xenobiotics (7%).

**Figure 4:**
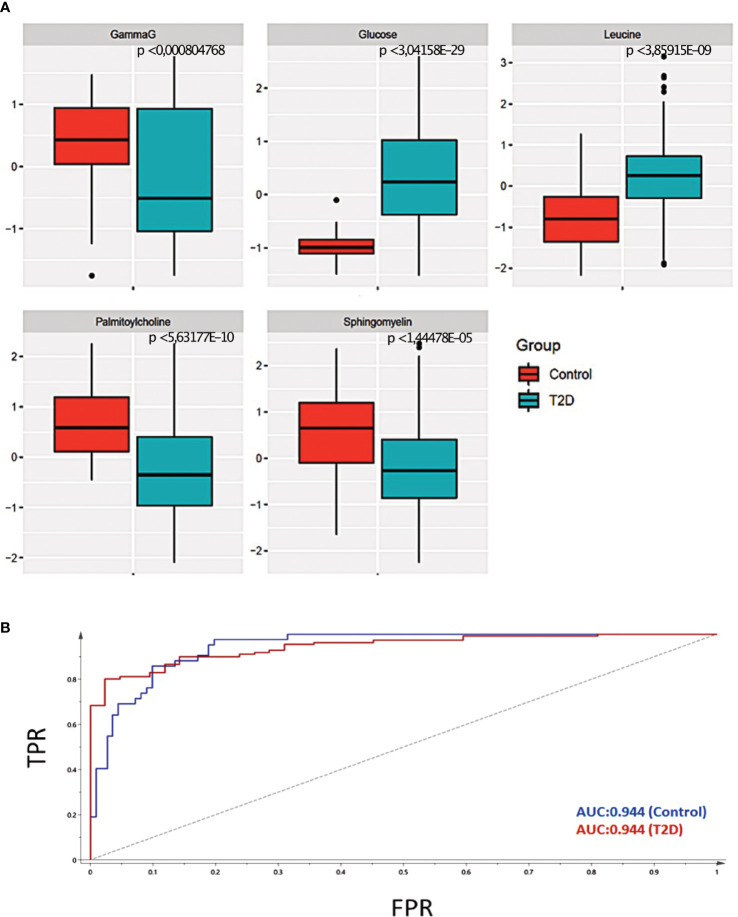
Selection of representative metabolites for data integration. **(A)** Statistical significance of the five selected metabolites for data integration. The p-value is estimated using an unpaired t-test. The p-value < 0.05 is considered to the significant. **(B)** The Area Under the Curve (AUC) of the five selected metabolites was 0.94, demonstrating a high accuracy of prediction.

To characterize metabolic changes that are associated with T2D development in adolescents, we performed UPLC-MS/MS on the serum of fasted individuals. We initially identified a total of 820 individual metabolites. After the missing value estimation features with more than 20% missing values were removed, then after preprocessing we obtained 481 metabolites (**Supplementary Figure 1A**). A VIP score cutoff of >1.5 resulted in 43 significant metabolites (**Table 2**). To show the most significant super pathways for the significant metabolites, we plotted a fold change vs p-value volcano plot (**Supplementary Figure 5A**) and a frequency bar plot (**Supplementary Figure 5B**). We used an unsupervised approach and performed PCA analysis where the T2D adolescents were clearly separated from the control group (**Figure 1A**). The permutation plot of the PCA (**Figure 1B**) validates the robustness of the model. Further we used an orthogonal partial least discriminant analysis (OPLS-DA) predictive model with 43 metabolites and the variation explained or goodness of fit in control vs. T2D patients (R^2^) of 63.8% and a predictive variation or goodness of prediction (*Q* ^2^) had a value of 59.7% (**Supplementary Figure 3A**). To verify our model, we permuted the group labels (control and T2D patients) 100 times to generate random models and observed that our model was significantly different from the permuted variations (**Supplementary Figure 3B**). Differential levels of the 43 significant metabolites between control and T2D patient samples are shown in (**Figure 2**). Next, we performed unsupervised hierarchical clustering for the 43 metabolites, which formed five different clusters. Each cluster represented metabolites that mostly belong to similar sub-pathways (**Figure 3A**). Broadly, these 43 metabolites were categorized into seven super pathways, including lipids (35%), peptides (26% gamma-glutamyl amino acids), amino acids (10%), carbohydrates (17%), nucleotides (2% purine metabolism), cofactors and vitamins (2% ascorbate and aldarate metabolism), and xenobiotics (7%) (**Figure 3B**). Based on their biological relevance to metabolic health in diabetes, we selected metabolites from most of the clusters (**Figure 3A**). The differential levels of the selected metabolites (gamma-glutamyl isoleucine, glucose, leucine, palmitoyl choline and sphingomyelin) were statistically significant (**Figure 4A**; p <0.05) and were further used to integrate with the epigenetic data. To understand the robustness and accuracy of the five selected metabolites we estimated the area under the curve (AUC), which was 0.944 for both controls and T2D, demonstrating the robustness of our model (**Figure 4B**).

**Table 2:** Significant Serum Metabolites in Youth with T2D.

Biochemical	Comp ID	Super Pathway	Sub Pathway	KEGG ID	HMDB ID	PUBCHEM	Log2(FC)	FDR
Pyroglutamine	46225	Amino Acid	Glutamate Metabolism	NA	NA	134508	-0.95336	1.11E^-07^
Imidazole Lactate	15716	Amino Acid	Histidine Metabolism	C05568	HMDB02320	440129	-0.54818	2.98E^-08^
Leucine	60	Amino Acid	Leucine, Isoleucine and Valine Metabolism	C00123	HMDB00687	6106	0.29615	3.06E^-07^
Cystine	56	Amino Acid	Methionine, Cysteine, SAM and Taurine Metabolism	C00491	HMDB00192	67678	0.85621	0.017982
N-Acetyltaurine	48187	Amino Acid	Methionine, Cysteine, SAM and Taurine Metabolism	NA	NA	159864	-0.93978	3.07E^-16^
Creatinine	513	Amino Acid	Creatine Metabolism	C00791	HMDB00562	588	-0.23991	0.000023
Gamma-Glutamylalanine	37063	Peptide	Gamma-glutamyl Amino Acid	NA	HMDB29142	440103	-0.66669	0.000081
Gamma-Glutamylglutamate	36738	Peptide	Gamma-glutamyl Amino Acid	C05282	HMDB11737	92865	-0.63692	0.004894
Gamma-Glutamylglutamine	2730	Peptide	Gamma-glutamyl Amino Acid	C05283	HMDB11738	150914	-0.68509	5.84E^-10^
Gamma-Glutamylglycine	33949	Peptide	Gamma-glutamyl Amino Acid	NA	HMDB11667	165527	-1.1881	2.0E^-08^
Gamma-Glutamylhistidine	18245	Peptide	Gamma-glutamyl Amino Acid	NA	NA	7017195	-0.74326	0.000024
Gamma-Glutamylisoleucine	34456	Peptide	Gamma-glutamyl Amino Acid	NA	HMDB11170	14253342	-0.39281	0.020489
Gamma-Glutamyl-Alpha-Lysine	55015	Peptide	Gamma-glutamyl Amino Acid	NA	NA	65254	-0.74009	0.000010
Gamma-Glutamylmethionine	44872	Peptide	Gamma-glutamyl Amino Acid	NA	HMDB29155	7009567	-1.0817	1.74E^-09^
Gamma-Glutamylthreonine	33364	Peptide	Gamma-glutamyl Amino Acid	NA	HMDB29159	76078708	-0.72121	0.000049
Gamma-Glutamylvaline	43829	Peptide	Gamma-glutamyl Amino Acid	NA	HMDB11172	7015683	-0.49642	0.008625
Gamma-Glutamylserine	54914	Peptide	Gamma-glutamyl Amino Acid	NA	NA	22844748	-0.68682	0.000036
1,5-Anhydroglucitol (1,5-AG)	20675	Carbohydrate	Glycolysis, Gluconeogenesis, and Pyruvate Metabolism	C07326	HMDB02712	64960	-1.997	9.46E^-20^
Glucose	48152	Carbohydrate	Glycolysis, Gluconeogenesis, and Pyruvate Metabolism	C00031	HMDB00122	79025	0.95686	2.62E^-14^
Ribonate	27731	Carbohydrate	Pentose Metabolism	C01685	HMDB00867	5460677	1.0173	2.31E^-09^
Fructose	577	Carbohydrate	Fructose, Mannose and Galactose Metabolism	C00095	HMDB00660	5984	0.99634	3.68E^-09^
Mannose	48153	Carbohydrate	Fructose, Mannose and Galactose Metabolism	C00159	HMDB00169	18950	1.0482	1.41E^-12^
N-Acetyl-glucosamine/N-Acetylgalactosamine	46539	Carbohydrate	Aminosugar Metabolism	NA	HMDB00215	24139	-0.27191	0.000042
Palmitoylcholine	52944	Lipid	Fatty Acid Metabolism (Acyl Choline)	NA	NA	151731	-1.0277	3.87E^-08^
Oleoylcholine	53260	Lipid	Fatty Acid Metabolism (Acyl Choline)	NA	NA	59040790	-1.0407	8.96E^-08^
Linoleoylcholine	57463	Lipid	Fatty Acid Metabolism (Acyl Choline)	NA	NA	NA	-1.1395	2.15E^-09^
Stearoylcholine	57464	Lipid	Fatty Acid Metabolism (Acyl Choline)	NA	NA	NA	-1.1569	0.000000044
Arachidonoylcholine	53261	Lipid	Fatty Acid Metabolism (Acyl Choline)	NA	NA	122198216	-1.2076	2.06E^-08^
1-(1-Enyl-Palmitoyl)-2-Palmitoleoyl-GPC (P-16:0/16:1)	52713	Lipid	Plasmalogen	NA	HMDB11207	52923882	-0.50345	1.16E^-10^
1-(1-Enyl-Palmitoyl) -2-Oleoyl-GPC (P-16:0/18:1)	52478	Lipid	Plasmalogen	NA	NA	NA	-0.34017	2.49E^-06^
Sphingomyelin (D18:2/14:0, D18:1/14:1)	47154	Lipid	Sphingolipid Metabolism	NA	NA	NA	-0.38072	0.000207
Sphingomyelin (D18:1/20:1, D18:2/20:0)	48491	Lipid	Sphingolipid Metabolism	NA	NA	NA	-0.31732	1.00E^-06^
Sphingomyelin (D18:2/24:1, D18:1/24:2)	52437	Lipid	Sphingolipid Metabolism	NA	NA	NA	-0.25523	0.000010
Sphingomyelin (D18:2/23:1)	57482	Lipid	Sphingolipid Metabolism	NA	NA	NA	-0.34705	0.000085
Sphingomyelin (D18:1/20:2, D18:2/20:1, D16:1/22:2)	57481	Lipid	Sphingolipid Metabolism	NA	NA	NA	-0.40633	0.000085
Sphingomyelin (D18:2/20:2/24:2)	57479	Lipid	Sphingolipid Metabolism	NA	NA	NA	-0.51498	3.37E^-10^
Sphingomyelin (D18:1/22:2, D18:2/22:1, D16:1/24:2)	57477	Lipid	Sphingolipid Metabolism	NA	NA	NA	-0.42945	2.98E^-07^
Glycosyl Ceramide (D18:2/24:1, D18:1/24:2)	57453	Lipid	Ceramides	NA	NA	NA	-0.44786	8.52E^-07^
7-Methylguanine	35114	Nucleotide	Purine Metabolism, Guanine containing	C02242	HMDB00897	11361	-0.2625	0.000006
Oxalate (Ethanedioate)	20694	Cofactors and Vitamins	Ascorbate and Aldarate Metabolism	C00209	HMDB02329	971	1.2738	0.000087
Gluconate	587	Xenobiotics	Food Component/Plant	C00257	HMDB00625	10690	1.2306	1.16E^-10^
2-Keto-3-Deoxy-Gluconate	48141	Xenobiotics	Food Component/Plant	C00204	HMDB01353	161227	1.2781	6.48E^-10^
Tartronate (Hydroxymalonate)	20693	Xenobiotics	Bacterial/Fungal	C02287	HMDB35227	45	-0.96185	0.000006

NA, not available; FC, fold change; FDR, False discovery rate; Comp ID, Compound ID.

The super and the sub pathways of metabolites determined by their KEGG, HMDB and, PUBCHEM ids. The folds changes are converted to log2 values. FDR calculated unpaired t-test. The negative sign indicates the lower levels and positive value is higher levels as compared to the controls.

### Differential methylation of DNA in adolescents with T2D

To describe the epigenetic changes associated with adolescents with T2D in First Nations youth, we performed DNA methylation profiling on PBMC genomic DNA from the iCARE cohort study participants. The bioinformatic pipeline used to obtain the significant DMRs is shown in (**Supplementary Figure 1B**). After peak calling and peak filtering, we obtained 732984 peaks. Using linear regression comparing adolescents with and without T2D, we controlled for age, sex, BMI and surrogate variables to correct for cell type differences, we obtained 459 significant peaks (**Supplementary Table 1**). Peaks were annotated to cis-regulatory regions of the TSS of the nearest genes (-5000 kb upstream to 5000 kb downstream). Using the above criteria, we obtained 42 regions out of which 36 significant DMRs were within the 5kb upstream or downstream window of the TSS and six were close to more than one gene (**Table 3**). Among these 36 DMRs seven were located near the TSS. Some of these genes have biological relevance to T2D, such as *FFAR1*, *USF2*, *C1QTNF9*, Arylsulfatase A (*ARSA*), Chromodomain Helicase DNA Binding Protein 8 (*CHD8*), Protocadherin Alpha 1 (*PCDHA1*) and Natriuretic Peptide B (*NPPB*) (**Table 3**). The methylation peaks of the nearest DMRs to the *FFAR1*, *C1QTNF9* and *USF2* genes are represented in **Supplementary Figures 6A–C**).

**Table 3:** Significant peaks that are differentially methylated between T2D and controls.

Chromosome	Start	End	Peak number	Nearest gene	Distance from TSS	Methylation Status
chr22	51064477	51064877	Peak117	*ARSA*	1923	Decreased
chr8	22412013	22412413	Peak135	*SORBS3*	3005	Decreased
chr11	67810920	67811320	Peak137	*TCIRG1*	4637	Decreased
chr1	11919744	11920144	Peak16	*NPPB*	-956	Increased
chr19	38016396	38016796	Peak166	*ZNF793*	2289	Increased
chr1	27189779	27190179	Peak176	*SFN*	346	Decreased
chr4	190943560	190943960	Peak18	*FRG2*	4652	Decreased
chr19	3767151	3767551	Peak184	*MRPL54*	4689	Decreased
chr19	3767151	3767551	Peak184	*RAX2*	4882	Decreased
chr19	4557053	4557766	Peak185	*SEMA6B*	2410	Decreased
chr16	68269125	68269525	Peak190	*ESRP2*	1162	Decreased
chr20	61923486	61923886	Peak205	*COL20A1*	-852	Decreased
chr11	823636	824345	Peak21	*EFCAB4A*	-3594	Decreased
chr19	35843080	35843480	Peak271	*FFAR1*	835	Decreased
chr11	56057882	56058282	Peak272	*OR8H1*	484	Decreased
chr14	21899880	21900280	Peak275	*CHD8*	-213	Decreased
chr11	840258	840658	Peak289	*POLR2L*	2087	Decreased
chr11	840258	840658	Peak289	*TSPAN4*	-3988	Decreased
chr12	108083057	108083457	Peak310	*PWP1*	3748	Increased
chr12	51478875	51479275	Peak320	*CSRNP2*	-1742	Increased
chr22	30727674	30728074	Peak338	*TBC1D10A*	-4984	Increased
chr11	67191345	67191745	Peak34	*RPS6KB2*	-4427	Decreased
chr6	24723903	24724303	Peak380	*C6ORF62*	-3039	Decreased
chr11	46726740	46727140	Peak381	*ARHGAP1*	-4791	Decreased
chr11	46726740	46727140	Peak381	*ZNF408*	4572	Decreased
chr19	1065608	1066008	Peak404	*HMHA1*	-114	Decreased
chr17	79782003	79782403	Peak42	*FAM195B*	2335	Decreased
chr13	24882421	24882821	Peak428	*C1QTNF9*	1317	Decreased
chr16	58030446	58031214	Peak432	*USB1*	-4447	Decreased
chr16	58030446	58031214	Peak432	*ZNF319*	2932	Decreased
chr6	30587385	30587785	Peak435	*MRPS18B*	2099	Increased
chr6	30587385	30587785	Peak435	*PPP1R10*	-2564	Increased
chr19	35757516	35757916	Peak44	*USF2*	-2252	Decreased
chr2	18765729	18766129	Peak458	*NT5C1B*	4883	Decreased
chr9	139835090	139835490	Peak51	*C8G*	-4423	Decreased
chr9	139835090	139835490	Peak51	*FBXW5*	3812	Decreased
chr5	140167607	140168007	Peak64	*PCDHA1*	1931	Decreased
chr20	39990179	39990942	Peak75	*EMILIN3*	4906	Decreased
chr2	241512174	241512574	Peak76	*RNPEPL1*	4270	Decreased
chr19	11642058	11642458	Peak77	*ECSIT*	-2269	Decreased
chr13	21349929	21350329	Peak81	*N6AMT2*	-2041	Increased
chr17	48613911	48614311	Peak87	*EPN3*	4207	Decreased

All the significant peaks that were annotated using GREAT -5000 to 5000 bp of the TSS. Chromosome indicates the chromosome number of the peak, start indicates the start of peak, end indicates the end of peak, peak number is the unique number of a peak, nearest gene indicates the closest gene to the center of the peak, Distance from TSS indicates the distance of the peak from the TSS of the gene. The methylation status is indicated as increased or decreased.

### Correlation between metabolites and DNA methylation in youth with T2D

**Figure 5:**
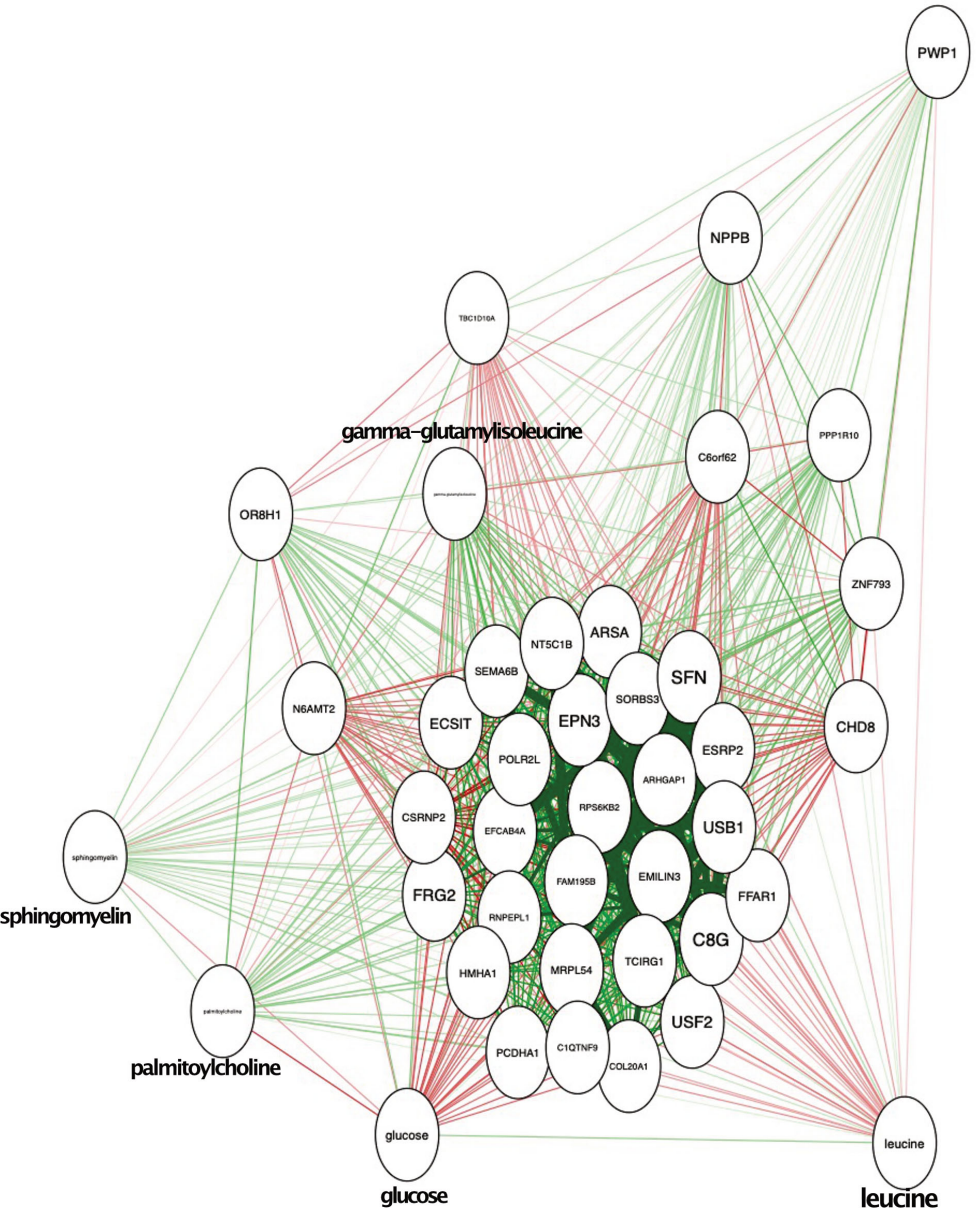
Integration of metabolic and epigenomic data. The red lines indicate the positive correlation and green lines indicate the negative correlation. The five metabolites and the genes are shown in the circles.

To improve our understanding of mechanisms involved in metabolic perturbations in youth-onset T2D, we investigated the link between DNA methylation and altered levels of metabolites. To predict the T2D status using the selected 5 metabolites the value of the Area Under the Curve (AUC) was 0.94, demonstrating a high accuracy of prediction (**Figure 4B**). We used a graph-based correlation method to find the significant correlations between the five representative metabolites and the 36 DMRs that were located within the -5kb and 5kb window of the TSS of the nearest genes. **Figure 5** shows that upon data integration, the metabolites were negatively (green lines) as well as positively correlated (red lines) to several genes. Glucose was correlated with 31 DMRs, leucine correlated to 7 DMRs, gamma-glutamylisoleucine correlated with 30 DMRs, palmitoylcholine was correlated with 10 DMRs and sphingomyelin was correlated to a single DMR (**Supplementary Table 2**). Interestingly, *FFAR1* was negatively correlated to glucose and leucine, but was positively correlated to gamma-glutamylisoleucine. *USF2* was also positively correlated with gamma-glutamylisoleucine and negatively correlated with leucine and glucose. *C1QTNF9* was positively correlated to gamma-glutamylisoleucine and negatively correlated to glucose. We further correlated all the metabolites and 36 DMRs and found several of them to be highly correlated (**Supplementary Figure 4**). The number of DMRs that correlated to metabolites are shown in **Supplementary Table 4**.

## Discussion

T2D in adolescents is aggressive and phenotypically different from T2D in adults (4, 23). The mechanisms underlying these differences are poorly understood although detrimental environmental exposures related to poverty, food insecurity, and poor housing related to the impact of colonization likely have an important role (24). To our knowledge this is the first study to link differential DNA methylation in PBMCs with the serum metabolome in youth-onset T2D. Using stringent VIP scores, we identified 43 metabolites associated with T2D and by peak calling for adolescents with T2D vs the controls, we obtained 459 significant peaks. Among these 459 DMRs, 36 were located near the TSS of genes. Some of these DMRs correlated with the selected metabolites, including 31 that were associated with fasting glucose levels, 7 correlated with leucine, 30 with gamma-glutamyl isoleucine and 10 with palmitoyl choline. DMRs that strongly associated with several of the metabolites in T2D patients included DMRs near the TSS in *FFAR1*, *USF2*, and *C1QTNF9*. Interestingly, these three genes have biological relevance in T2D. This data highlights that complementary information provided by epigenetic marks provide new insight into the metabolic perturbations occurring in adolescents with T2D. Future research will examine the novel role for the DMRs near these genes in regulating gene expression and serum metabolite levels in T2D.

We found that amino acids were the most commonly altered metabolite in the circulation of adolescents with T2D. In adults, high levels of aromatic and BCAAs are predictive of future T2D development (25) and a strong negative association exists between these levels and insulin sensitivity (26, 27). Altered amino acid catabolism in adipose tissue is believed to be the underlying reason that amino acid levels are altered in obese and insulin resistant adults (28). In adolescents, elevated levels of BCAAs have been reported to be associated with obesity (11) and impaired diastolic cardiac function (13). Unlike adults, increased levels of BCAAs were positively associated with beta-cell function, relative to insulin sensitivity in adolescents (8, 10). Consistent with these findings we observed that the BCAA leucine was increased in the serum of adolescents with T2D. Given that seven DMRs correlated with serum leucine levels in T2D, this finding sets the stage to examine whether these DMRs are involved in regulating leucine levels in adolescents that could underlie the differential effects of BCAAs on beta-cell function in adolescents and adults with T2D. Notably, levels of a broad range of gamma-glutamyl dipeptides were also reduced in adolescents with T2D. Gamma-glutamyl amino acids are considered to be involved in regulating oxidative stress through their involvement in glutathione production.

We identified an association between lipid metabolism and adolescents with T2D. Reductions in circulating levels of several sphingomyelins, lysophosphatidylcholines and acyl-alkyl phosphatidylcholine (plasmalogens) were observed. These acyl-alkyl phosphatidylcholines belong to a class of antioxidant plasmalogens and could reflect the state of oxidative stress. On the other hand, since lysophosphatidylcholines in the bloodstream are derived from oxidation of phosphatidylcholine in low density lipoproteins, could suggests a reduction in its oxidation. These results are consistent with a previous study showing that serum levels of acyl-alkyl phosphatidylcholines and lysophosphatidylcholines are reduced in obese children (29). In adult populations, elevated sphingolipids are generally associated with obesity and greater insulin resistance (30). We observed reductions in a number of sphingolipids in First Nations adolescents with T2D. This finding is consistent with another study of normoglycemic North American Indigenous adolescents and young adults (31) that identified an association between obesity and lowered sphingolipid. Thus, there appears to be a role for altered lipid metabolism in the natural history of T2D. In light of this, nutritional strategies developed by and for First Nations people should be crucial to improving their health status as a whole (32).

We identified a DMR near *FFAR1* that was positively correlated with gamma-glutamylisoleucine and negatively correlated with glucose and leucine. *FFAR1* (also known as *GPR40*) induces the G_αq_ signaling cascade which activates phospholipase C and inositol 1,4,5-triphosphate (IP3) formation, stimulating Ca^2+^ mobilization from the endoplasmic reticulum and triggering insulin secretion (33). Free fatty acids are proposed to potentiate glucose-stimulated insulin secretion through FFAR1 activation, evidenced by a reduction in Ca^2+^ oscillations following the inactivation of FFAR1 using GW1100 (a GPR40 inhibitor) (34). Given that *FFAR1* mRNA and protein expression are reduced in the islets of diabetic mice (35) and an FFAR1 agonist improved glucose and lipid metabolism in obese mice (36), it is conceivable that altered DNA methylation near the TSS of the *FFAR1* gene could be associated with alterations in insulin secretion and glucose homeostasis in adolescents with T2D, although this hypothesis requires further investigation.

Another relevant discovery was the identification of a DMR near the TSS of the *USF2* gene that correlated with glucose, leucine and gamma-glutamylisoleucine. USF2 is a ubiquitous basic helix-loop-helix transcription factor that binds to E-box elements. High glucose levels upregulate *USF2* expression in the liver and USF2 regulates SREBP-1c and stimulates fatty acid synthesis in the liver that leads to lipid accumulation (37, 38). This is consistent with our previous finding that hepatic steatosis was 3-fold higher in First Nation adolescents with T2D compared to normoglycemic controls (39), although whether methylation of the *USF2* promoter is a contributing factor remains to be investigated.

We also identified a DMR near the TSS of the *C1QTNF9* gene, that was correlated with serum glucose and gamma-glutamylisoleucine levels in adolescents with T2D. *C1QTNF9* encodes a novel cytokine, termed CTRP9, that is a paralog of adiponectin and is expressed by adipose tissue, heart and endothelium. CTRP9 protects cells against high glucose and palmitate-induced oxidative stress (40, 41). CTRP9 has been reported to attenuate diabetic nephropathy and improve cardiac function in obese and diabetic mice (42, 43). However, increased CTRP9 in the circulation correlated with insulin resistance in humans (44, 45), suggesting that more studies investigating CTRP9 actions in T2D are necessary. Given that Dart et al. (46, 47) reported a higher incidence and earlier onset of major diabetes-related complications in a cohort of adolescents with T2D compared to a cohort of adolescents with type 1 diabetes, follow-up studies will examine the association between alterations in DNA methylation and the risk for complications of diabetes in youth. These findings correspond with a growing body of literature linking genome-wide alterations in DNA methylation to complications of diabetes (48, 49).

In Canada, First Nation youth account for a disproportionate number of T2D diagnoses (50). Environmental influences, including nutrition, have a major role in defining T2D risk, but T2D in youth is also associated with poverty and lower socioeconomic status (47, 48, 51). Epigenetic changes mediate environmental influences on the genetic architecture. We uncovered several DNA methylation and metabolite alterations that provide important new knowledge about the cellular changes occurring in First Nations adolescents associated with T2D. This supports the theory that social inequities, purposeful starvation, dispossession of land and traditional ways of living in First Nations youth induced molecular and biological manifestations of chronic disease risk (49). However, we also acknowledge that our study cannot separate whether these changes in metabolites and DNA methylation are a consequence of T2D development or contribute to the development of T2D in youth. One of the major limitations of our analysis is the small sample size. Since our study only captures a snapshot of the changes following a diagnosis of T2D, further studies are warranted in a larger sample size in the iCARE cohort, as well as replication in other populations to determine whether these findings are generalizable to the wider population of adolescents with T2D or whether these changes are unique to First Nation adolescents. Future work in longitudinal settings would also provide a clearer picture of the mechanisms involved in the development of T2D and its associated complications in adolescents. Finally, we recognize that the serum metabolomic profile may not be reflective of the metabolic changes in all tissues. Moreover, the DNA methylation patterns in PBMCs may not reflect methylation and gene expression changes in all tissues that generate serum metabolites. However, some of the genes and metabolites have been separately linked to altered metabolism in T2D by previous studies, suggesting that our integrative approach provides relevant information about youth-onset T2D. Nonetheless, future functional studies determining mechanisms of how DNA methylation induces changes in gene expression and the observed metabolites are warranted.

In summary, we integrated serum metabolomic and genome-wide DNA methylation data in a systems medicine-based approach that is well suited to generate new hypotheses about the complex and variable factors that contribute to the development of T2D in pediatric populations. We identified several robust candidate DMRs located near the TSS of genes such as *USF2*, *FFAR1* and *C1QTNF9* that are correlated to a collection of metabolites that are relevant to metabolic homeostasis in T2D. We will test the hypothesis that T2D induced DNA methylation of *USF2*, *FFAR1* and *C1QTNF9*, among others, affect gene expression and metabolite levels. This study lays the groundwork for future research about how epigenetic and metabolite alterations relate to T2D pathogenesis in adolescents and evaluate their predictive power in the development of T2D and its associated complications. Our findings generate new hypotheses that will test whether the identified DMRs regulate the expression of the nearby genes in metabolic cell types and whether altered expression of these genes influences metabolite levels. Elucidating the linkages between DNA methylation patterns in PBMCs and circulating metabolites would alsoserum highlight the advantage of integrating data from multiple sources.

## Data availability statement

The DNA methylation and metabolomic data presented in the study are deposited in the European Genome-phenome Archive database (EGAS00001003816). The remaining data are available from the corresponding author upon reasonable request and approval by the iCARE data access committee.

## Ethics statement

The studies involving human participants were reviewed and approved by the University of Manitoba/Health Sciences Research ethics board. The iCARE cohort study (15) received informed consent from study participants and approval from the University of Manitoba/Health Sciences Centre Research Ethics Board (HS13255), First Nations patient and parent advisory committee and the First Nation Health and Social Secretariat of Manitoba and the iCARE participant and parent advisory committees. Written informed consent to participate in this study was provided by the participants’ legal guardian/next of kin.

## Author contributions

PA, MJ and AA conceived and designed the data analysis strategy and integration. MF prepared clinical samples for analysis. PA, NH, CT and AA performed data analysis. VWD, MJ and JD provided oversight, analysis and coordination of all aspects listed above. BW, AD, ES and JM developed the iCARE cohort study, sample collection and clinical analyses. PA, BW, MJ, AA and VWD wrote the manuscript. All authors edited and reviewed the manuscript. VWD is the guarantor of this work and, as such, had full access to all data in the study and takes responsibility for the integrity of the data and the accuracy of the data analysis. All authors contributed to the article and approved the submitted version.

## Funding

This research is supported by a CIHR grant (MOP#142309) to AD and BW and an Environments, Genes and Chronic Disease CIHR Team Grant #144626 to VWD, BW, JD et al. Funding sources were not involved in the study design, collection, interpretation of the data or preparation of the manuscript.

## Acknowledgments

The authors are grateful to Dr. Wanda Phillips-Beck of Nanaandawewigamig First Nations Health and Social Secretariat of Manitoba for providing comments about this paper from an Indigenous perspective. PA was the recipient of a postdoctoral fellowship from Research Manitoba. JMM holds an Applied Public Health Chair awarded by the Canadian Institutes for Health Research (CIHR) #CPP-137910. JRD was a Canada Research Chair (Tier 1) in Chromatin Dynamics. AA is supported by the National Institute for Health Research (NIHR) Surgical Reconstruction and Microbiology Research Centre (SRMRC), Birmingham, UK. VWD was the Allen Rouse-Manitoba Medical Services Foundation Basic Scientist. The views expressed in this publication are those of the authors and not necessarily those of the National Health Service (NHS), the National Institute for Health Research (NIHR), UK.

## Conflict of interest

The authors declare that the research was conducted in the absence of any commercial or financial relationships that could be construed as a potential conflict of interest.

## Publisher’s note

All claims expressed in this article are solely those of the authors and do not necessarily represent those of their affiliated organizations, or those of the publisher, the editors and the reviewers. Any product that may be evaluated in this article, or claim that may be made by its manufacturer, is not guaranteed or endorsed by the publisher.

## Supplementary material

Supplementary Figure 1 Schematic workflow of the analysis for epigenetic and metabolomic datasets. **(A)** Shows the workflow of the data processing steps, normalization and, data analysis steps followed for obtaining the significant metabolites. **(B)** Shows the workflow of data processing, normalization and, analysis to obtain the significant DMRs.

Supplementary Figure 2 Pearson correlation between all the clinical variables of primary cohort. **(A)** Clinical variables of controls that include age, height, weight, waist size, BMI, glucose levels, AST, ALT and HbA1c levels. Similarly, **(B)** Clinical variables of T2D patients.

Supplementary Figure 3 Serum metabolomic profile of youth with T2D. **(A)** OPLSDA model based on the significant metabolites (i.e. 43 metabolites). The controls are shown in green and the T2D samples are in blue. **(B)** Permutation conducted to validate the variation obtained during the OPLSDA. The R2 (shown in green) and Q2 (shown in blue) values indicate the robustness of the OPLSDA model.

Supplementary Figure 4 Correlation plot for all the significant metabolites and 36 DMRs. Red color shows the positively correlated and blue shows negatively correlated. Non correlated ones are shown in white. The five metabolites chosen for data integration are indicated by a red arrow.

Supplementary Figure 5 Clusters of super pathways for metabolites. a) x-axis of the volcano plot for the super pathways shows the log2 fold change and y-axis shows the p-value. b) x-axis of the bar plot shows the super pathways, and the y-axis shows the frequency of each pathway.

Supplementary Figure 6 Visualization of DMRs genomic location using IGV genome viewer. Red color is for the 10 control samples and blue color for 21 T2D samples. All the shown regions have decreased methylation as compared to controls. The two vertical lines in each figure shows the approx. peak region. **(A)** shows genomic location of DMR nearest to TSS of the gene FFAR1. **(B)** shows genomic location of the DMR nearest to TSS of gene C1QTNF9. **(C)** shows genomic location of the DMR nearest to TSS of the gene USF2.

Supplementary Table 1 Significant DMRs/peaks file. The table contains all the significant DMRs/peaks obtained in the DNA methylation analysis. The file contains the genomic location of each peak followed by the significance level.

Supplementary Table 2 Data integration correlation values. The table contains the correlation values obtained from the DMR and five metabolites integration. The green colored values show the negative correlation and red values show the positive correlation.

Supplementary Table 3 Information of the DNA methylation sequencing samples. The table provides the information of the samples that were sequenced and submitted in the European Genome-phenome Archive.

Supplementary Table 4 All the correlated metabolites and DMRs
