# Supplementary material for: Integrative analysis reveals novel associations between DNA methylation and the serum metabolome of adolescents with type 2 diabetes: A cross-sectional study
Source: Front Endocrinol (Lausanne). 2022 Oct 10;13:934706. doi: 10.3389/fendo.2022.934706 (PMC9593237; doi:10.3389/fendo.2022.934706)
Supplement: Supplementary file 1 [file DataSheet_1.docx]

**Online Supplemental Information**

**Integrative analysis reveals novel associations between DNA methylation and the serum metabolome of adolescents with type 2 diabetes: A cross-sectional study.**

Prasoon Agarwal^1,2^, Brandy A. Wicklow^1,3^, Allison B. Dart^1,3^, Nikho A. Hizon^1,4^, Elizabeth A.C. Sellers^1,3^, Jonathan M. McGavock^1,3^, Charlotte P. J. Talbot ^1,2^, Mario A. Fonseca^1,2^, Wayne Xu^4,5^, James R. Davie^1,4,5^, Meaghan J. Jones^1,4^, Animesh Acharjee^6, 7, 8*^, and Vernon W. Dolinsky^1,2*^

^1^Diabetes Research Envisioned and Accomplished in Manitoba (DREAM), Research Theme of the Children’s Hospital Research Institute of Manitoba,^2^Department of Pharmacology and Therapeutics, ^3^Department of Pediatrics and Child Health, ^4^Department of Biochemistry and Medical Genetics, ^5^Research Institute in Oncology and Hematology, ^6^Institute of Cancer and Genomic Sciences University of Birmingham, ^7^Institute of Translational Medicine, University Hospitals Birmingham NHS Foundation Trust, Birmingham, B15 2TT, UK, ^8^NIHR Surgical Reconstruction and Microbiology Research Centre, Birmingham, B15 2TT, UK

*Sharing senior authors for correspondence

Dr. Vernon W. Dolinsky

e-mail: vdolinsky@chrim.ca

Associate Professor of Pharmacology & Therapeutics

Co-lead of the Diabetes Research Envisioned and Accomplished in Manitoba (DREAM) Research Theme of the Children’s Hospital Research Institute of Manitoba.

601 John Buhler Research Centre,

715 McDermot Avenue

University of Manitoba,

Winnipeg, MB, Canada R3E 3P4

Telephone number: +1 (204) 789-3559

Fax Number: 204-789-3915

Dr. Animesh Acharjee

e-mail: a.acharjee@bham.ac.uk

Institute of Cancer and Genomic Sciences University of Birmingham, B15 2TT, UK

Institute of Translational Medicine, University Hospitals Birmingham NHS Foundation Trust, Birmingham, B15 2TT, UK

NIHR Surgical Reconstruction and Microbiology Research Centre, Birmingham, B15 2TT, UK

Telephone number: +44 (0) 1213718135

**Metabolomics analysis of T2D adolescent serum**

**Sample Preparation**: Samples were prepared using the automated MicroLab STAR® system (Hamilton Company, Boston, U.S.A).   To remove protein, dissociate small molecules bound to protein or trapped in the precipitated protein matrix, and to recover chemically diverse metabolites, proteins were precipitated with methanol under vigorous shaking for 2 min using Geno Grinder 2000 (Glen Mills Inc., NJ, U.S.A) followed by centrifugation.  The resulting extract was divided into five fractions: two for analysis by two separate reverse phase (RP)/UPLC-MS/MS methods with positive ion mode electrospray ionization (ESI), one for analysis by RP/UPLC-MS/MS with negative ion mode ESI, one for analysis by HILIC/UPLC-MS/MS with negative ion mode ESI, and one sample was reserved for backup. Samples were placed briefly on a TurboVap® (Zymark, MA, U.S.A) to remove the organic solvent.  The sample extracts were stored overnight under nitrogen before preparation for analysis.

**Ultrahigh Performance Liquid Chromatography-Tandem Mass Spectroscopy (UPLC-MS/MS):**  All methods utilized a Waters ACQUITY ultra-performance liquid chromatography (UPLC) and a Thermo Scientific Q-Exactive high resolution/accurate mass spectrometer interfaced with a heated electrospray ionization (HESI-II) source and Orbitrap mass analyzer operated at 35,000 mass resolution. The sample extract was dried then reconstituted in solvents compatible to each of the four methods. Each reconstitution solvent contained a series of standards at fixed concentrations to ensure injection and chromatographic consistency. One aliquot was analyzed using acidic positive ion conditions, chromatographically optimized for more hydrophilic compounds. In this method, the extract was gradient eluted from a C18 column (Waters UPLC BEH C18-2.1x100 mm, 1.7 µm) using water and methanol, containing 0.05% perfluoropentanoic acid (PFPA) and 0.1% formic acid (FA).  Another aliquot was also analyzed using acidic positive ion conditions, however it was chromatographically optimized for more hydrophobic compounds. In this method, the extract was gradient eluted from the same aforementioned C18 column using methanol, acetonitrile, water, 0.05% PFPA and 0.01% FA and was operated at an overall higher organic content. Another aliquot was analyzed using basic negative ion optimized conditions using a separate dedicated C18 column. The basic extracts were gradient eluted from the column using methanol and water, however with 6.5mM Ammonium Bicarbonate at pH 8. The fourth aliquot was analyzed via negative ionization following elution from a HILIC column (Waters UPLC BEH Amide 2.1x150 mm, 1.7 µm) using a gradient consisting of water and acetonitrile with 10mM Ammonium Formate, pH 10.8. The MS analysis alternated between MS and data-dependent MS^n^ scans using dynamic exclusion.  The scan range varied slighted between methods but covered 70-1000 m/z.

**Data Extraction and Compound Identification:** Raw data was extracted, peak-identified and QC processed using Metabolon’s hardware and software. Compounds were identified by comparison to library entries of purified standards or recurrent unknown entities.  Metabolon maintains a library based on authenticated standards that contains the retention time/index (RI), mass to charge ratio (*m/z)*, and chromatographic data (including MS/MS spectral data) on all molecules present in the library.  Furthermore, biochemical identifications are based on three criteria: retention index within a narrow RI window of the proposed identification, accurate mass match to the library +/- 10 ppm, and the MS/MS forward and reverse scores between the experimental data and authentic standards.  The MS/MS scores are based on a comparison of the ions present in the experimental spectrum to the ions present in the library spectrum. While there may be similarities between these molecules based on one of these factors, the use of all three data points can be utilized to distinguish and differentiate biochemicals.  More than 3300 commercially available purified standard compounds have been acquired and registered into LIMS for analysis on all platforms for determination of their analytical characteristics. Additional mass spectral entries have been created for structurally unnamed biochemicals, which have been identified by virtue of their recurrent nature (both chromatographic and mass spectral).  These compounds have the potential to be identified by future acquisition of a matching purified standard or by classical structural analysis.

**Metabolite Quantification and Data Scaling**

MetaboAnalyst4.0 (1) was used to filter and scale the metabolomics data. Peaks were quantified using area-under-the-curve. Before data analysis, a data integrity check is performed to make sure that all the necessary information has been collected. The class labels must be present and contain only two classes. Using the default parameters, first the features were removed that were missing > 20% in all the samples then and all the missing values were replaced with a small value (the half of the minimum positive value in the original data) assuming to be the detection limit. The assumption of this approach is that most missing values are caused by low abundance metabolites (i.e. below the detection limit). Further, data filtration was done without using the phenotype information using the Interquartile Range (IQR) method thus obtained 481 metabolites. The data was transformed and scaled using the cube root and mean centering respectively. Further, we selected the 5 metabolites based on their biological properties and from each cluster obtained in figure 1d. The selection was made based on previous knowledge and role of these metabolites in the disease biology.

**DNA Methylation data preprocessing and bioinformatics analysis**

The 50-bp SOLiD single end sequence reads were ensured by quality check (noise to signal ratio). The sequence reads were mapped on the human reference genome (hg19) using the MethylMiner™ Mapping Analysis module of the LifeScope v2.5.1 software package (Life Technologies). We performed two stages of mapping with the second stage of gap alignments. A 2-scoring penalty was used for mismatches and bases with a quality value below 10 were replaced with ‘N’. We used mapping quality values for recognizing or filtering good alignments. A mapping quality value estimates the probability that the alignment is correct, and takes several indications into account, including the number of alignments or uniqueness of an alignment. We set a good quality score of 8 in BAM files. We used BamStats for mapping statistics and statistics on read coverage and depth. The mapped bam files were viewed using IGV or Partek Genomics Suite software (Partek Incorporated, St. Louis, Missouri U.S.A.). During the analysis, we excluded the X, Y and, M (mitochondrial DNA) chromosomes to avoid sex differences during our analysis.

**References:**

1. Xia J, Psychogios N, Young N, Wishart DS: MetaboAnalyst: a web server for metabolomic data analysis and interpretation. Nucleic Acids Res 2009;37:W652-660
