# Supplementary figures and images for: Integrative analysis reveals novel associations between DNA methylation and the serum metabolome of adolescents with type 2 diabetes: A cross-sectional study
Source: Front Endocrinol (Lausanne). 2022 Oct 10;13:934706. doi: 10.3389/fendo.2022.934706 (PMC9593237; doi:10.3389/fendo.2022.934706)

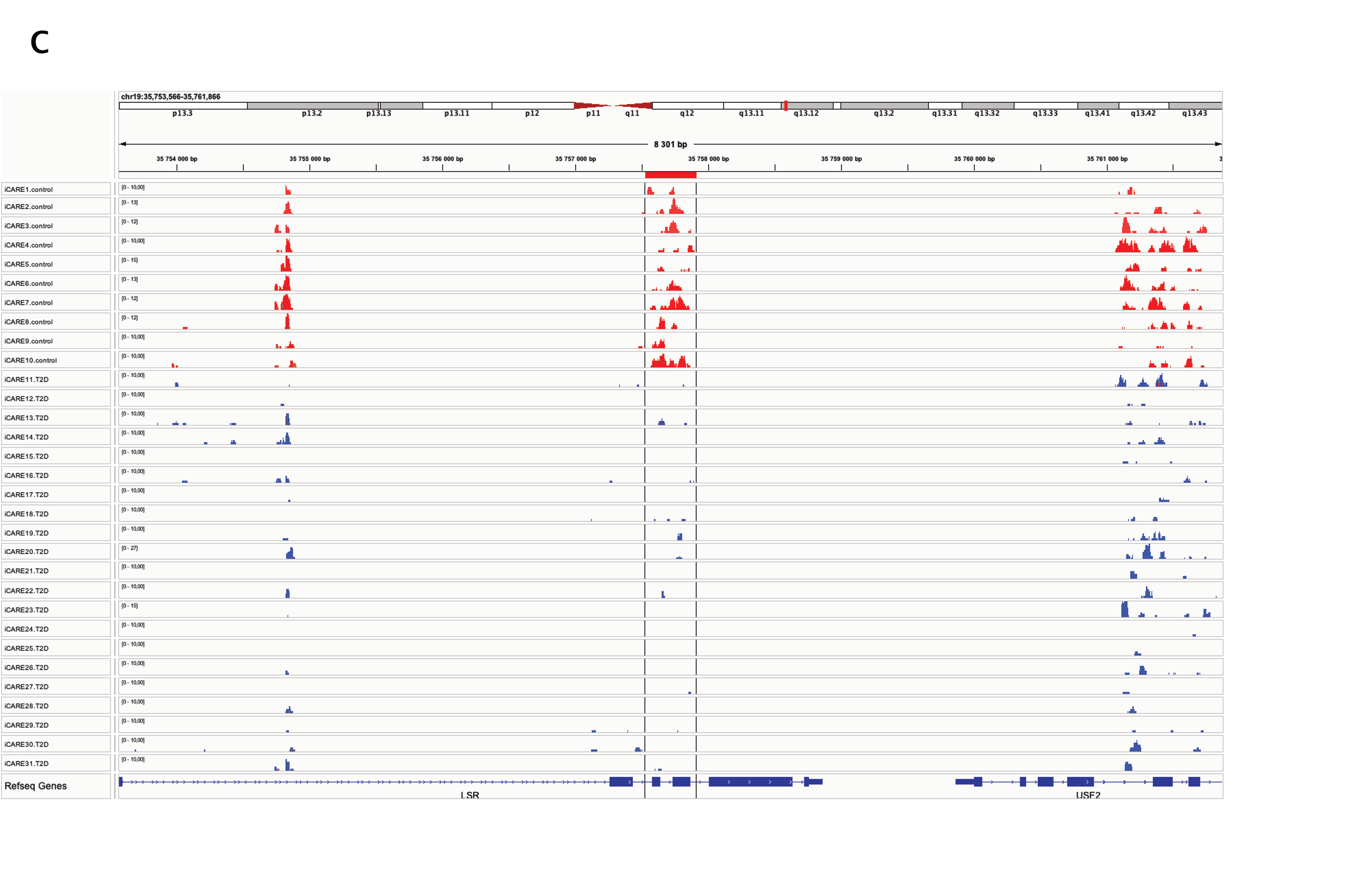

Supplement: Supplementary file 2 [file Image_7.tiff]

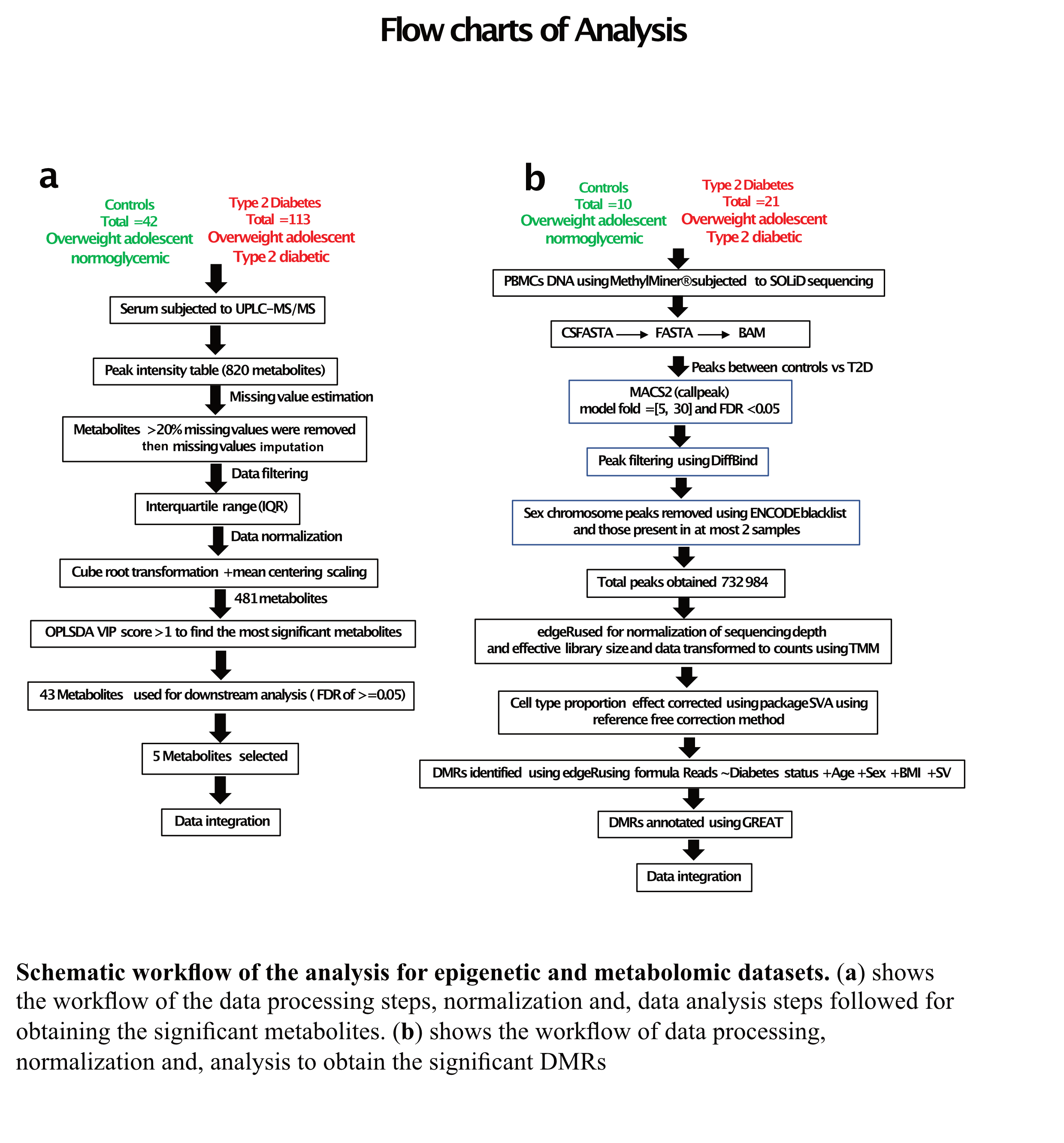

Supplement: Supplementary Figure 1 — Schematic workflow of the analysis for epigenetic and metabolomic datasets. (A) Shows the workflow of the data processing steps, normalization and, data analysis steps followed for obtaining the significant metabolites. (B) Shows the workflow of data processing, normalization and, analysis to obtain the significant DMRs. [file Image_1.tiff]

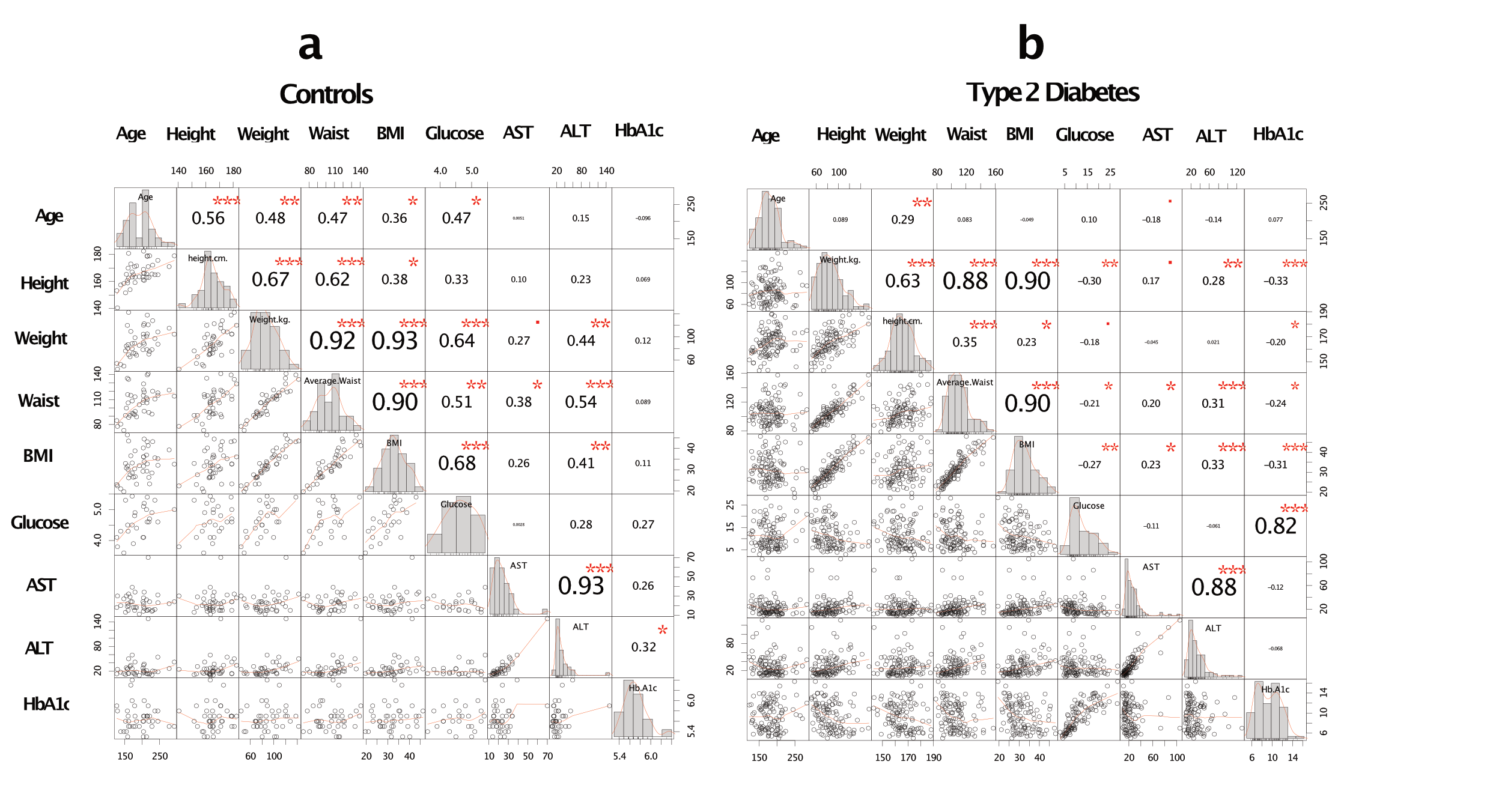

Supplement: Supplementary Figure 2 — Pearson correlation between all the clinical variables of primary cohort. (A) Clinical variables of controls that include age, height, weight, waist size, BMI, glucose levels, AST, ALT and HbA1c levels. Similarly, (B) Clinical variables of T2D patients. [file Image_2.tiff]

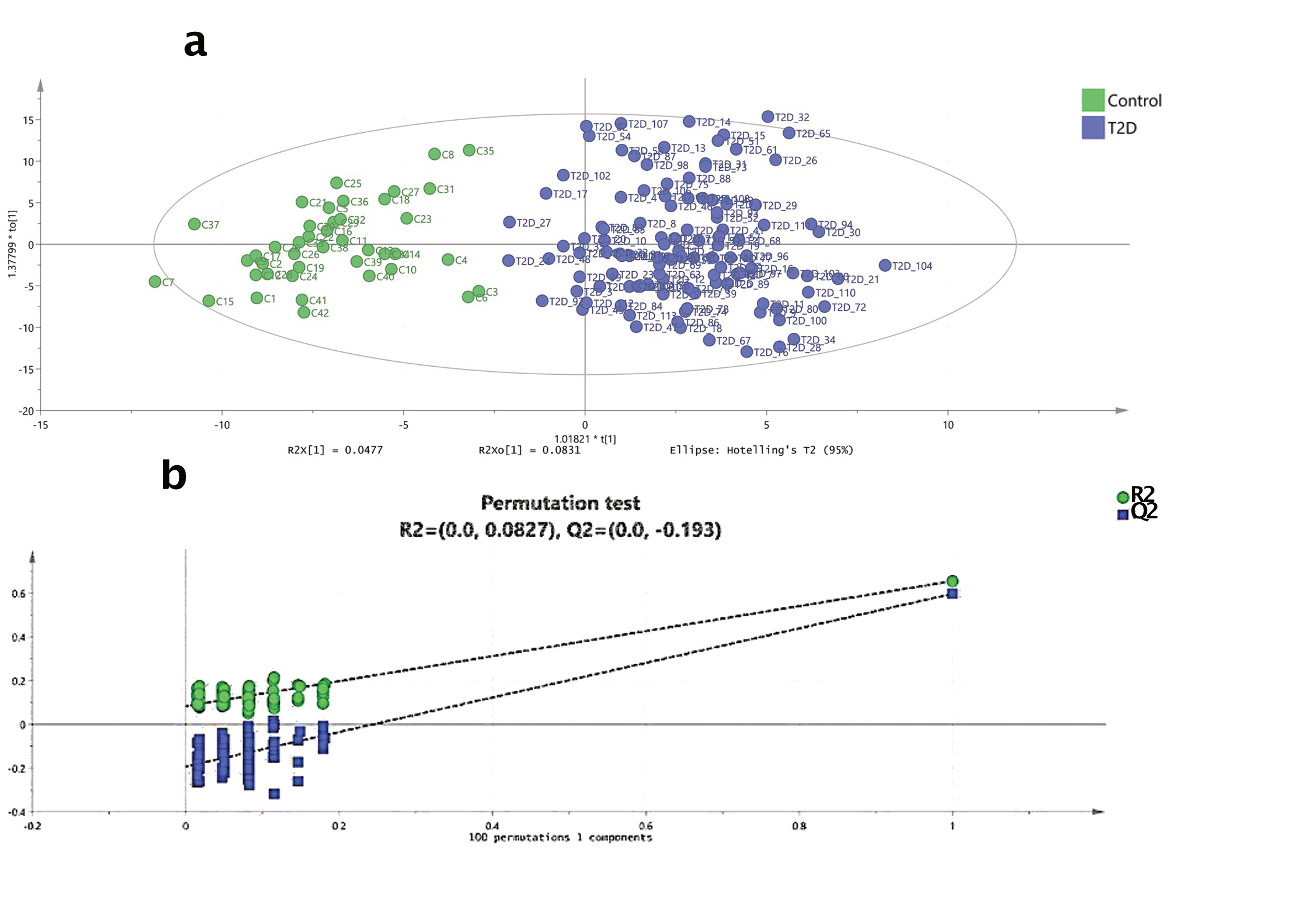

Supplement: Supplementary Figure 3 — Serum metabolomic profile of youth with T2D. (A) OPLSDA model based on the significant metabolites (i.e. 43 metabolites). The controls are shown in green and the T2D samples are in blue. (B) Permutation conducted to validate the variation obtained during the OPLSDA. The R2 (shown in green) and Q2 (shown in blue) values indicate the robustness of the OPLSDA model. [file Image_3.tiff]

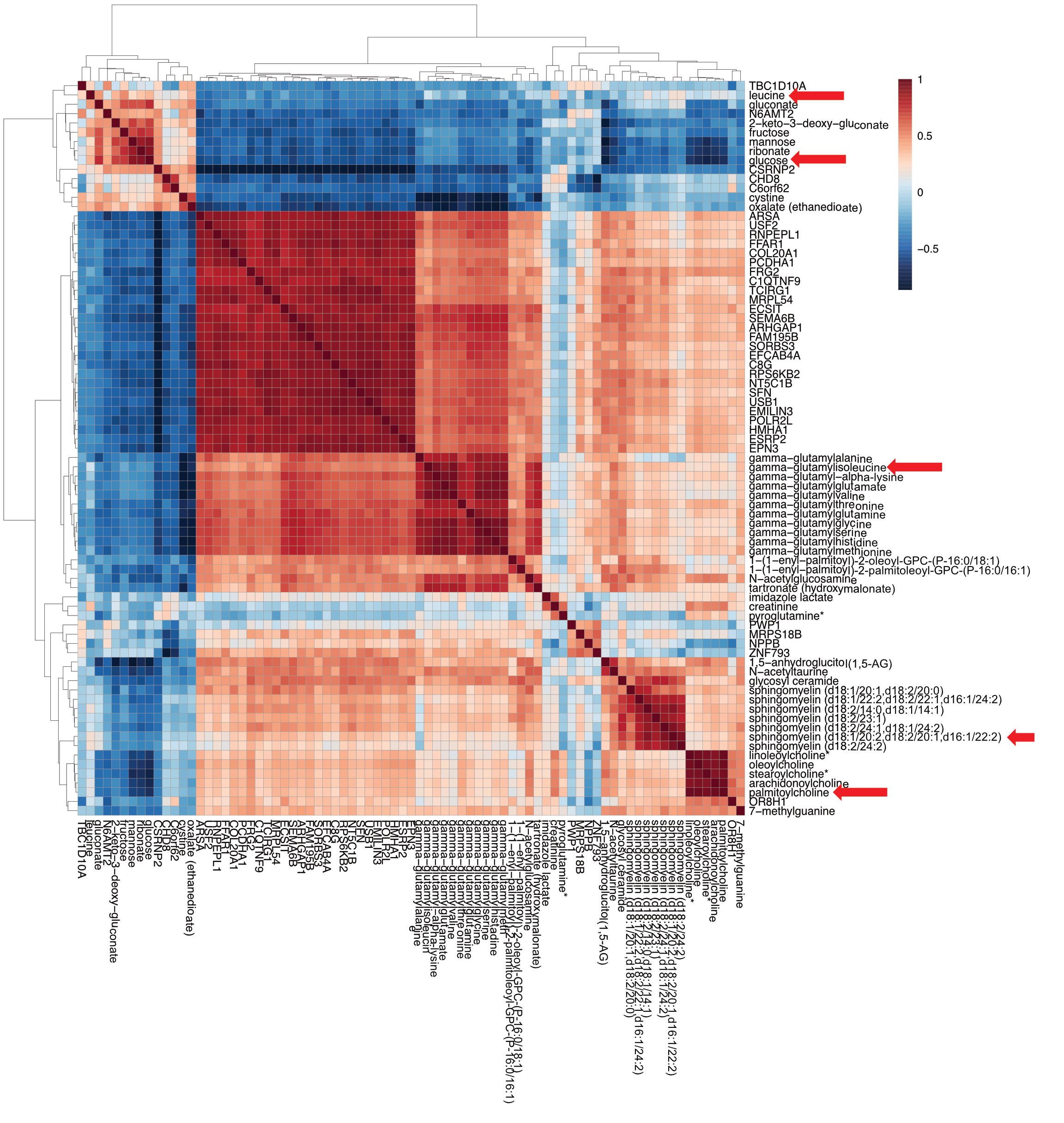

Supplement: Supplementary Figure 4 — Correlation plot for all the significant metabolites and 36 DMRs. Red color shows the positively correlated and blue shows negatively correlated. Non correlated ones are shown in white. The five metabolites chosen for data integration are indicated by a red arrow. [file Image_4.tiff]

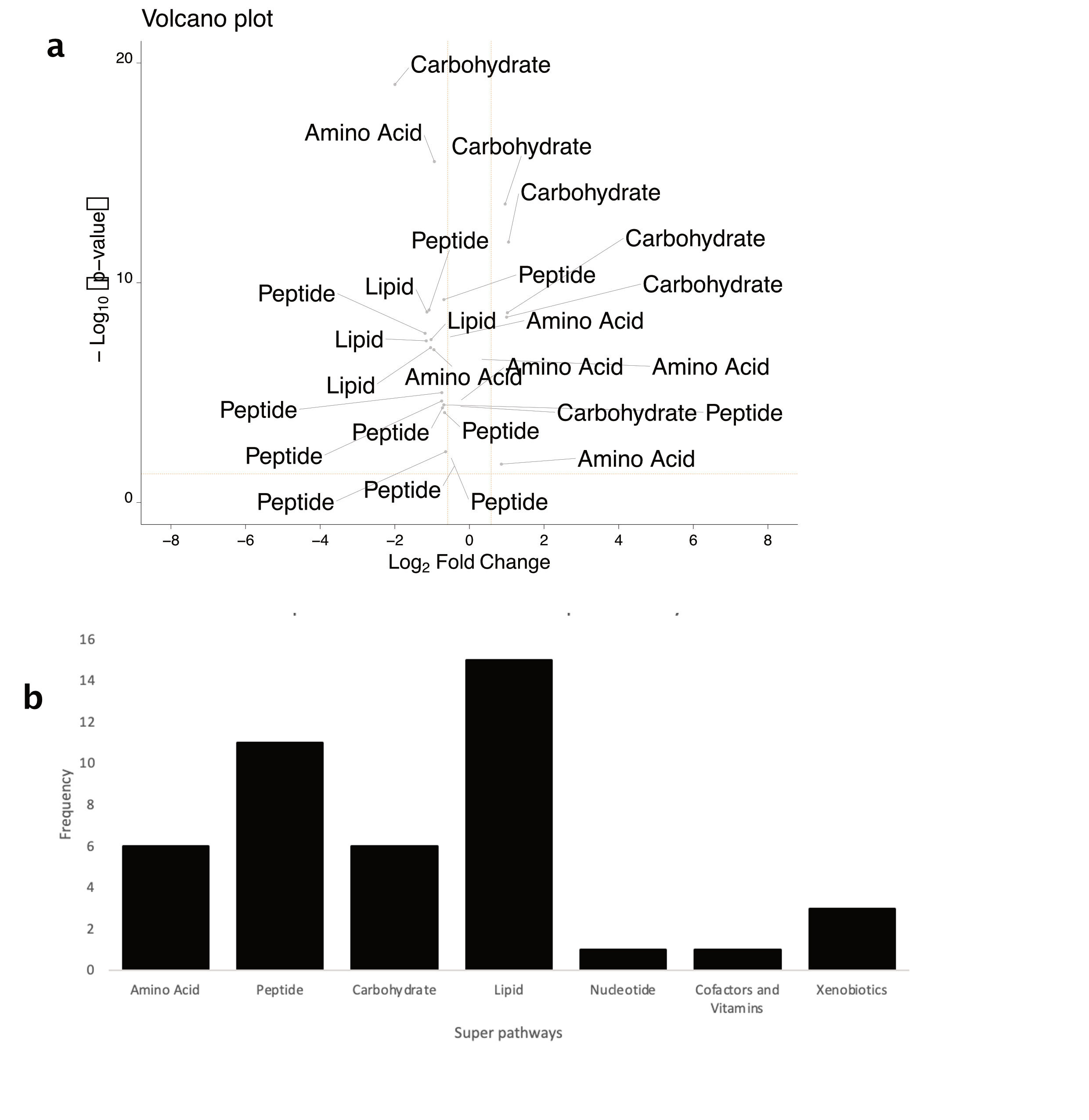

Supplement: Supplementary Figure 5 — Clusters of super pathways for metabolites. a) x-axis of the volcano plot for the super pathways shows the log2 fold change and y-axis shows the p-value. b) x-axis of the bar plot shows the super pathways, and the y-axis shows the frequency of each pathway. [file Image_5.tiff]

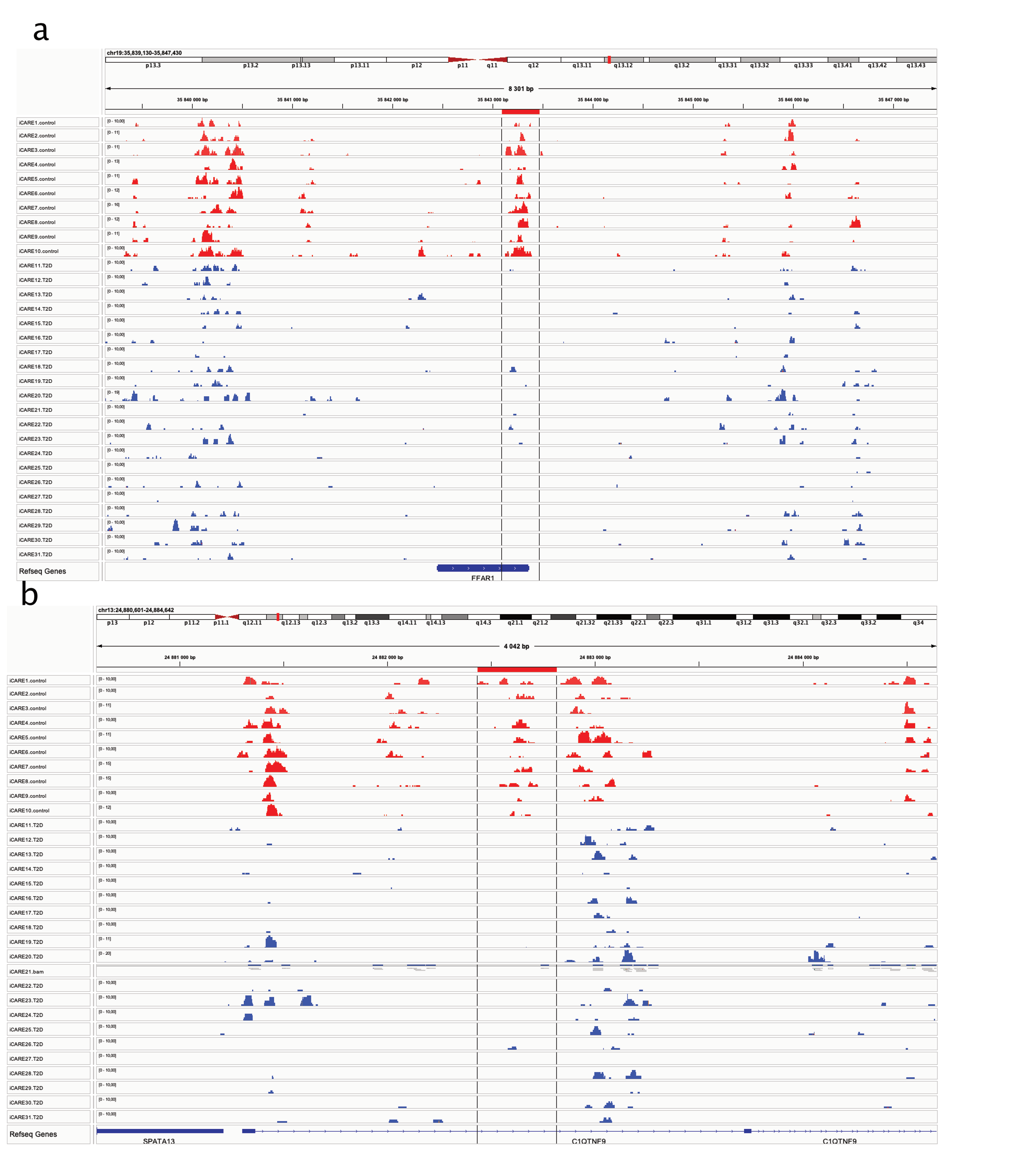

Supplement: Supplementary Figure 6 — Visualization of DMRs genomic location using IGV genome viewer. Red color is for the 10 control samples and blue color for 21 T2D samples. All the shown regions have decreased methylation as compared to controls. The two vertical lines in each figure shows the approx. peak region. (A) shows genomic location of DMR nearest to TSS of the gene FFAR1. (B) shows genomic location of the DMR nearest to TSS of gene C1QTNF9. (C) shows genomic location of the DMR nearest to TSS of the gene USF2. [file Image_6.tiff]
