# Supplementary material for: Integrative analysis reveals novel associations between DNA methylation and the serum metabolome of adolescents with type 2 diabetes: A cross-sectional study
Source: Front Endocrinol (Lausanne). 2022 Oct 10;13:934706. doi: 10.3389/fendo.2022.934706 (PMC9593237; doi:10.3389/fendo.2022.934706)
Supplement: Supplementary Table 3 — Information of the DNA methylation sequencing samples. The table provides the information of the samples that were sequenced and submitted in the European Genome-phenome Archive. [file Table_3.docx]

| EGA submitted IDs | Alias | description | subjectId | gender | Cell type | region | phenotype |
| --- | --- | --- | --- | --- | --- | --- | --- |
| iCARE1 | control1 | "Control sample" | C1 | male | PBMCs | Canada | Non diabetic |
| iCARE2 | control2 | "Control sample" | C2 | female | PBMCs | Canada | Non diabetic |
| iCARE3 | control3 | "Control sample" | C3 | female | PBMCs | Canada | Non diabetic |
| iCARE4 | control4 | "Control sample" | C4 | male | PBMCs | Canada | Non diabetic |
| iCARE5 | control5 | "Control sample" | C5 | female | PBMCs | Canada | Non diabetic |
| iCARE6 | control6 | "Control sample" | C6 | male | PBMCs | Canada | Non diabetic |
| iCARE7 | control7 | "Control sample" | C7 | female | PBMCs | Canada | Non diabetic |
| iCARE8 | control8 | "Control sample" | C8 | female | PBMCs | Canada | Non diabetic |
| iCARE9 | control9 | "Control sample" | C9 | female | PBMCs | Canada | Non diabetic |
| iCARE10 | control10 | "Control sample" | C10 | female | PBMCs | Canada | Non diabetic |
| iCARE11 | T2D_1 | "T2D sample" | IC1 | female | PBMCs | Canada | Type 2 Diabetes |
| iCARE12 | T2D_2 | "T2D sample" | IC2 | female | PBMCs | Canada | Type 2 Diabetes |
| iCARE13 | T2D_3 | "T2D sample" | IC3 | male | PBMCs | Canada | Type 2 Diabetes |
| iCARE14 | T2D_4 | "T2D sample" | IC4 | female | PBMCs | Canada | Type 2 Diabetes |
| iCARE15 | T2D_5 | "T2D sample" | IC5 | female | PBMCs | Canada | Type 2 Diabetes |
| iCARE16 | T2D_6 | "T2D sample" | IC6 | male | PBMCs | Canada | Type 2 Diabetes |
| iCARE17 | T2D_7 | "T2D sample" | IC7 | female | PBMCs | Canada | Type 2 Diabetes |
| iCARE18 | T2D_8 | "T2D sample" | IC8 | female | PBMCs | Canada | Type 2 Diabetes |
| iCARE19 | T2D_9 | "T2D sample" | IC9 | female | PBMCs | Canada | Type 2 Diabetes |
| iCARE20 | T2D_10 | "T2D sample" | IC10 | male | PBMCs | Canada | Type 2 Diabetes |
| iCARE21 | T2D_11 | "T2D sample" | IC11 | male | PBMCs | Canada | Type 2 Diabetes |
| iCARE22 | T2D_12 | "T2D sample" | IC12 | female | PBMCs | Canada | Type 2 Diabetes |
| iCARE23 | T2D_13 | "T2D sample" | IC13 | female | PBMCs | Canada | Type 2 Diabetes |
| iCARE24 | T2D_14 | "T2D sample" | IC14 | male | PBMCs | Canada | Type 2 Diabetes |
| iCARE25 | T2D_15 | "T2D sample" | IC15 | female | PBMCs | Canada | Type 2 Diabetes |
| iCARE26 | T2D_16 | "T2D sample" | IC16 | female | PBMCs | Canada | Type 2 Diabetes |
| iCARE27 | T2D_17 | "T2D sample" | IC17 | female | PBMCs | Canada | Type 2 Diabetes |
| iCARE28 | T2D_18 | "T2D sample" | IC18 | male | PBMCs | Canada | Type 2 Diabetes |
| iCARE29 | T2D_19 | "T2D sample" | IC19 | female | PBMCs | Canada | Type 2 Diabetes |
| iCARE30 | T2D_20 | "T2D sample" | IC20 | male | PBMCs | Canada | Type 2 Diabetes |
| iCARE31 | T2D_21 | "T2D sample" | IC21 | female | PBMCs | Canada | Type 2 Diabetes |

Supplementary Table 3
