# Supplementary material for: Integrative analysis reveals novel associations between DNA methylation and the serum metabolome of adolescents with type 2 diabetes: A cross-sectional study
Source: Front Endocrinol (Lausanne). 2022 Oct 10;13:934706. doi: 10.3389/fendo.2022.934706 (PMC9593237; doi:10.3389/fendo.2022.934706)
Supplement: Supplementary Table 4 — All the correlated metabolites and DMRs [file Table_4.docx]

**Supplementary Table 4:**

| **Metabolites** | **No of correlated DMRs** |
| --- | --- |
| gamma-glutamylalanine | 31 |
| gamma-glutamyl.alpha-lysine | 31 |
| gamma-glutamylglycine | 31 |
| gamma-glutamylhistidine | 31 |
| gamma-glutamylmethionine | 31 |
| gamma-glutamylvaline | 31 |
| gamma-glutamylserine | 32 |
| gamma-glutamylglutamine | 30 |
| gamma-glutamylglutamate | 30 |
| cysteine | 30 |
| gamma-glutamylthreonine | 29 |
| 1-(1-Enyl-Palmitoyl)-2-Palmitoleoyl-GPC (P-16:0/16:1) | 28 |
| 1-(1-Enyl-Palmitoyl)-2-Oleoyl-GPC (P-16:0/18:1) | 28 |
| glycosyl-ceramide (D18:2/24:1, D18:1/24:2) | 28 |
| linoleoylcholine | 11 |
| oleoylcholine | 21 |
| arachidonoylcholine | 14 |
| stearoylcholine | 14 |
| sphingomyelin (D18:1/20:1, D18:2/20:0 | 18 |
| sphingomyelin (D18:2/24:1, D18:1/24:2) | 4 |
| sphingomyelin (D18:2/14:0, D18:1/14:1) | 27 |
| sphingomyelin (D18:2/23:1) | 21 |
| sphingomyelin (D18:2/24:1, D18:1/24:2) | 17 |
| sphingomyelin (D18:2/24:2) | 23 |
| mannose | 28 |
| fructose | 28 |
| gluconate | 16 |
| 1,5-Anhydroglucitol | 29 |
| 2-keto-3-deoxy-gluconate | 28 |
| N-acetylglucosamine-N-acetylgalactosamine | 29 |
| 7-methylguanine | 12 |
| ribonate | 28 |
| creatinine | 1 |
| N.acetyltaurine | 27 |
| tartronate.hydroymalonate | 28 |
| oalate-ethanedioate(oxalate | 30 |
